# Expanding the genetic spectrum of hereditary spherocytosis: novel mutations and phenotypic heterogeneity from a 55-patient cohort

**DOI:** 10.1007/s00277-026-07066-1

**Published:** 2026-05-13

**Authors:** Huiying Shu, Liqing Yang, Yu Gao, Xuexue Yun, Min Zhou, Yuping Gong

**Affiliations:** 1 Department of Hematology & Oncology, Chengdu Women’s and Children’s Central Hospital, University of Electronic Science and Technology of China, Chengdu, Sichuan Province 611731 China; 2 Fujian Provincial Key Laboratory on Hematology, Fujian Institute of Hematology, Fujian Medical University Union Hospital, Fuzhou, Fujian Province 350001 China; 3 Department of Hematology, West China Hospital, Sichuan University, Chengdu, Sichuan Province 610041 China

**Keywords:** Hereditary spherocytosis, NGS, Clinical manifestation, Gene mutation

## Abstract

To characterize the clinical and genetic profiles of patients with hereditary spherocytosis (HS) and report novel mutations. This retrospective study included 55 patients (25 males, 30 females; median age 9 years) with HS admitted between 2016 and 2023. Clinical and laboratory data were analyzed. Targeted next-generation sequencing was performed to detect pathogenic mutations. Comparisons were made using Student’s *t*-test or Mann-Whitney *U* test. Anemia was present in 53 patients, including 31 with moderate-to-severe anemia; 40 exhibited splenomegaly or gallstones. A total of 60 variants were identified, of which 46 (76.7%) were novel. The most frequently involved genes were *ANK1* (28, 50.9%), *SPTB* (17, 30.9%), *SLC4A1* (10, 18.2%), and *SPTA1* (2, 3.6%). Patients aged ≤ 3 years (*n* = 23) showed significantly lower red blood cell counts, hemoglobin levels, and absolute reticulocyte counts than those aged > 3 years (*n* = 32) (all *P* < 0.05), whereas no significant phenotypic differences were observed across mutation types. Younger patients with HS exhibited more severe anemia. *ANK1* and *SPTB* were the predominant pathogenic genes, with no notable phenotypic differences between these two genotypes. The identification of 46 novel mutations expands the mutational spectrum of HS.

**Supplementary Information:**

The online version contains supplementary material available at 10.1007/s00277-026-07066-1.

## Introduction

Hereditary spherocytosis (HS) is the most common congenital hemolytic anemia, resulting from defects in the erythrocyte membrane skeleton, and it can present at any age [1]. Common clinical manifestations include anemia, jaundice, splenomegaly, and an increased number of spherocytes in peripheral blood. Disease severity varies considerably, ranging from asymptomatic cases to life-threatening anemia [1]. HS is primarily caused by mutations in genes encoding red blood cell membrane proteins, including α-spectrin (*SPTA1*), β-spectrin (*SPTB*), ankyrin (*ANK1*), protein 4.2 (*EPB42*), and band 3 (*SLC4A1*) [2]. The condition is typically inherited in an autosomal dominant manner, although autosomal recessive cases have also been reported [3, 4]. Approximately 15–20% of HS cases are attributed to de novo pathogenic variants [5, 6]. These variants predominantly occur in the *ANK1* gene, a susceptibility likely driven by its high guanine-cytosine (G/C) content, which predisposes the locus to slipped-strand mispairing during DNA replication [7].

In this study, we retrospectively analyzed clinical data from 55 patients newly diagnosed with HS. Integrating next-generation sequencing (NGS) findings, we examined clinical manifestations and genetic mutation profiles across different age groups and genotypes. This work aims to advance the understanding of HS and inform clinical management by providing robust clinical and genetic evidence.

## Materials and methods

### Study design and patient population

This retrospective study analyzed data from 55 patients diagnosed with hereditary spherocytosis (HS) who were treated at Chengdu Women’s and Children’s Central Hospital and West China Hospital of Sichuan University between January 2016 and December 2023. The cohort comprised 25 males and 30 females, aged 0.75 to 22 years, with a median age of 9 years. Among the 55 patients, two were siblings (both carrying the *ANK1* c.1–16_6del22 mutation); all other patients were unrelated. Statistical comparisons between genotype groups were performed on unrelated patients only.

The diagnosis of HS was established based on the following criteria [8–10]: (1) family history of HS or clinical manifestations including anemia, jaundice, splenomegaly, or gallstones; (2) hematological findings demonstrating elevated reticulocyte counts and increased spherocytes on peripheral blood smears; (3) laboratory evidence of elevated indirect bilirubin levels, positive osmotic fragility test, and negative direct antiglobulin (Coombs) test; (4) identification of pathogenic mutations in HS-associated genes through genetic testing.

Exclusion criteria included glucose-6-phosphate dehydrogenase (G6PD) deficiency, thalassemia, unstable hemoglobinopathies, autoimmune hemolytic anemia, and other defined hemolytic anemias.

### Ethical considerations

This study was conducted in accordance with the Declaration of Helsinki and was approved by the Institutional Ethics Committee of Chengdu Women’s and Children’s Central Hospital and West China Hospital of Sichuan University. Written informed consent was obtained from all patients and/or their legal guardians prior to sample collection and genetic analysis.

### Genomic DNA extraction

Peripheral blood samples (2 mL) were collected from all patients and from available parents into EDTA-coated tubes. Genomic DNA was extracted using the Beijing Tian gen Peripheral Blood Genomic DNA Extraction Kit (Tian gen Biotech, Beijing, China), following the manufacturer’s instructions.

### Next-generation sequencing

Targeted high-throughput sequencing was performed by Wuhan Kang sheng da Medical Laboratory (Wuhan, China) using the MGISEQ-T7 platform (MGI Tech, Shenzhen, China). The custom-designed NGS panel covers approximately 700 genes associated with hereditary hematologic and immunologic disorders, including genes related to red blood cell membranopathies, red blood cell enzymopathies, congenital dyserythropoietic anemia, inherited bone marrow failure syndromes, and hemoglobinopathies. The panel targets all coding exons, exon-intron boundaries (± 20 bp), and selected regulatory regions. The average sequencing depth across all target regions was 500*–*1000×, with overall coverage exceeding 99%.

### Bioinformatics analysis

Raw sequencing data were processed through a standardized bioinformatics pipeline. Sequencing reads were aligned to the human reference genome (GRCh38) using the Burrows-Wheeler Aligner (BWA).

Variant annotation was performed using multiple databases, including 1000 Genomes Project, dbSNP, ClinVar, ESP6500, ExAC, Ensembl, HGMD and UCSC.

### Variant interpretation and classification

The pathogenic significance of identified variants was evaluated using multiple in silico prediction algorithms, including SIFT, PolyPhen-2, LRT, and Mutation Taster.

All identified variants were classified according to the American College of Medical Genetics and Genomics (ACMG) guidelines into five categories: pathogenic, likely pathogenic, variant of uncertain significance (VUS), likely benign, and benign. Clinical interpretation was performed by integrating genetic findings with patient phenotype, family history, and inheritance patterns.

### Methodological limitations

Several limitations of this targeted NGS approach should be acknowledged. First, this method primarily targets exon regions and is mainly applicable for point mutations and small insertions/deletions (≤ 20 bp), but is not suitable for detecting large copy number variations, dynamic mutations, complex rearrangements, genomic structural variants, large insertions, or mutations located in regulatory regions and introns. Second, due to inherent limitations of targeted capture methodology, detection cannot completely cover all designed probe regions, although overall coverage exceeds 99%. The presence of pseudogenes with high sequence similarity to disease-causing genes may lead to false-negative or false-positive results. Third, DNA used in this method was derived from patient blood or somatic cells rather than germ cells; therefore, mosaicism cannot be excluded. Fourth, due to current limitations in understanding of disease pathogenesis, many conditions have no identified causative genes or only partially reported causative genes. Failure to detect pathogenic mutations in specific genes does not exclude the possibility of disease. This detection technology carries a very small probability (< 5%) of error and requires comprehensive judgment in combination with other clinical manifestations and laboratory examinations.

### Statistical analysis

Statistical analyses were performed using SPSS version 19.0. Normally distributed continuous data are presented as mean±standard deviation (range), while non-normally distributed data are expressed as median (interquartile range). Categorical variables are reported as frequencies (percentages). Differences in clinical parameters between age groups and genotype categories were assessed using Student’s t-test or the Mann*-*Whitney U test, as appropriate. A two-tailed P value < 0.05 was considered statistically significant.

## Results

### General clinical characteristics of patients

The study included 55 patients diagnosed with HS, comprising 25 males and 30 females, with a median age at diagnosis of 9 years (range: 0.75*-*22 years). Among them, 53 (96.4%) exhibited anemia, including 15 cases of mild anemia (hemoglobin [Hb] 90*–*120 g/L), 21 cases of moderate anemia (Hb 60*–*90 g/L), and 10 cases of severe anemia (Hb < 60 g/L). Twenty-seven patients (49.1%) had a mean corpuscular hemoglobin concentration (MCHC) exceeding 340 g/L. The mean corpuscular volume (MCV) was 88.12 ± 7.92fL (range: 70.4*-*105.8fL), with 9 patients (16.4%) showing an MCV below 80fL. Additionally, 54 patients (98.2%) had a red blood cell distribution width*-*coefficient of variation (RDW-CV) greater than 14.5%, and 53 (96.4%) had absolute reticulocyte counts above 0.094 × 10¹²/L. All participants exhibited reticulocyte percentages greater than 1.5%. Total and indirect bilirubin levels were elevated (> 20.5 µmol/L) in 51 patients (92.7%). Furthermore, 40 patients (72.7%) presented with splenomegaly or cholelithiasis, as summarized in Table 1.

**Table 1:** Baseline demographic and clinical characteristics of 55 patients with HS at diagnosis

Variable	All patients (*n* = 55)	Normal reference range ^a^
Demographics		
Age, years, median (25*–*75% percentile)	9.00 (0.75*-*22.00)	—
Sex ratio (Male/Female)	1.2	—
Hematological Parameters		
RBC count, ×10¹²/L, mean ± SD (range)	2.85 ± 0.63 (1.28*–*4.26)	4.0*-*5.5 (Adults) 3.8*-*5.0 (Children)
Hemoglobin, g/L, mean ± SD (range)	85.55 ± 19.68 (49*–*127)	120*–*160 (M) 110*–*150 (F)
Hematocrit, %, median (25*–*75% percentile)	26.0(21.2*–*30)	40*–*50 (M) 37*–*47 (F)
MCV, fL, mean ± SD (range)	88.12 ± 7.92 (70.4*-*105.8)	80*–*100
MCHC, g/L, mean ± SD (range)	338.06 ± 18.32 (294*–*389)	320*–*360
Reticulocyte percentage, %, mean ± SD (range)	9.89 ± 4.47 (1.90*-*22.13)	0.5*–*1.5
Absolute reticulocyte count, ×10¹²/L, mean ± SD (range)	0.29 ± 0.13 (0.08*–*0.64)	0.02*–*0.08
Biochemical Parameters		
Total bilirubin, µmol/L, median (25*–*75% percentile)	65.40 (48.20*–*92.70)	3.4*–*20.5
Indirect bilirubin, µmol/L, median (25*–*75% percentile)	59.90 (41.30*–*82.90)	< 17.1
Clinical Complications, n (%)		
Splenomegaly and/or Cholecystolithiasis	40 (72.7%)^c^	—

Abbreviations: *RBC* red blood cell, *MCV* mean corpuscular volume, *MCHC* mean corpuscular hemoglobin concentration, *M* male, *F* female

Data are presented as mean±standard deviation (SD) with range, or median with interquartile range (25*–*75% percentile), or number (percentage) as appropriate

a Normal reference ranges are based on standard clinical laboratory values for adults and children (Henry’s Clinical Diagnosis and Management by Laboratory Methods, 24th ed.; WHO guidelines). Specific ranges may vary slightly by laboratory and age group

### Genetic analysis of patients

A total of 60 distinct variants were identified across four hereditary spherocytosis (HS)-associated genes: *ANK1*, *SPTB*, *SLC4A1*, and *SPTA1*. Of these, 46 (76.7%) were novel. At the patient level, 44 of 55 patients (80.0%) carried at least one novel variant. The distribution of gene involvement and variant types is summarized in Table 2.

*ANK1* was the most frequently involved gene, present in 28 patients (50.9%), either as a single gene or in combination with another HS-associated gene. *SPTB* was involved in 17 patients (30.9%), *SLC4A1* in 10 patients (18.2%), and *SPTA1* in 2 patients (3.6%). Frameshift variants were the most common overall (31.7%), followed by nonsense and missense variants (both 28.3%), and splice-site variants (11.7%). Notably, nonsense variants predominated in *SPTB* (47.1%) and missense variants predominated in *SLC4A1* (83.3%), whereas frameshift variants were most frequent in *ANK1* (53.6%).

**Table 2:** Gene involvement and variant type distribution among 55 patients with hereditary spherocytosis

Gene(s) involved	Patients, *n* (%)	95% CI	Total variants, *n*
*ANK1* alone	26 (47.3)	34.2–60.6	
*ANK1* + *SLC4A1*	1 (1.8)	0.1–9.9	
*ANK1* + *SPTB*	1 (1.8)	0.1–9.9	
Total *ANK1* involvement	**28 (50.9)**	**37.6–64.1**	**28**
*SPTB* alone	16 (29.1)	18.2–42.4	
*SPTB* + *ANK1*	1 (1.8)	0.1–9.9	
Total *SPTB* involvement	**17 (30.9)**	**19.6–44.6**	**17**
*SLC4A1* alone	7 (12.7)	6.8–26.4	
*SLC4A1* + *ANK1*	1 (1.8)	0.1–9.9	
*SLC4A1* compound heterozygous	2 (3.6)	0.6–12.5	
Total *SLC4A1* involvement	**10 (18.2)**	**9.5–30.6**	**12**
*SPTA1* alone	1 (1.8)	0.1–9.9	
*SPTA1* compound heterozygous	1 (1.8)	0.1–9.9	
Total *SPTA1* involvement	**2 (3.6)**	**0.6–12.5**	**3**
Overall	**55 (100)**	**—**	**60**

Table 3 presents the novelty, inheritance patterns, and pathogenicity classification of the identified variants. The highest proportion of novel variants was observed in ANK1 (92.9%) and SPTB (70.6%). Among cases with determined inheritance, de novo mutations accounted for 27.3% of variants, with the highest frequency in ANK1 (32.1%) and SPTB (29.4%). Based on ACMG/AMP guidelines, 38 of 60 variants (63.3%) were classified as pathogenic or likely pathogenic, while 21 variants (35.0%) were classified as variants of uncertain significance (VUS).

**Table 3:** Novelty, inheritance, and pathogenicity of variants by gene

Gene	Total variants	Novel variants, *n* (%)	De Novo mutations, *n* (%)	Inherited mutations, *n* (%)	Pathogenic/likely pathogenic variants, *n* (%)	VUS, *n* (%)
*ANK1*	28	26 (92.9)	9 (32.1)	19 (67.9)	**20 (71.4)**	**8 (28.6)**
*SPTB*	17	12 (70.6)	5 (29.4)	11 (68.7)	**12 (70.6)**	**5 (29.4)**
*SLC4A1*	12	7 (58.3)	1 (11.1)	8 (88.9)	**5 (41.7)**	**6 (50.0)**
*SPTA1*	3	1 (33.3)	0 (0.0)	2 (100.0)	**1 (33.3)**	**2 (66.7)**^
Total	**60**	**46 (76.7)**	**15 (27.3)** ^^a^^	**40 (72.7)** ^^a^^	**38 (63.3)** ^^b^^	**21(35.0)** ^^b^^

^a^ Percentages for de novo and inherited are calculated among cases with determined inheritance (ANK1: *n* = 28, SPTB: *n* = 17, SLC4A1: *n* = 12, SPTA1: *n* = 3)

^b^ Percentages are calculated based on total variants per gene

**Fig. 1:**
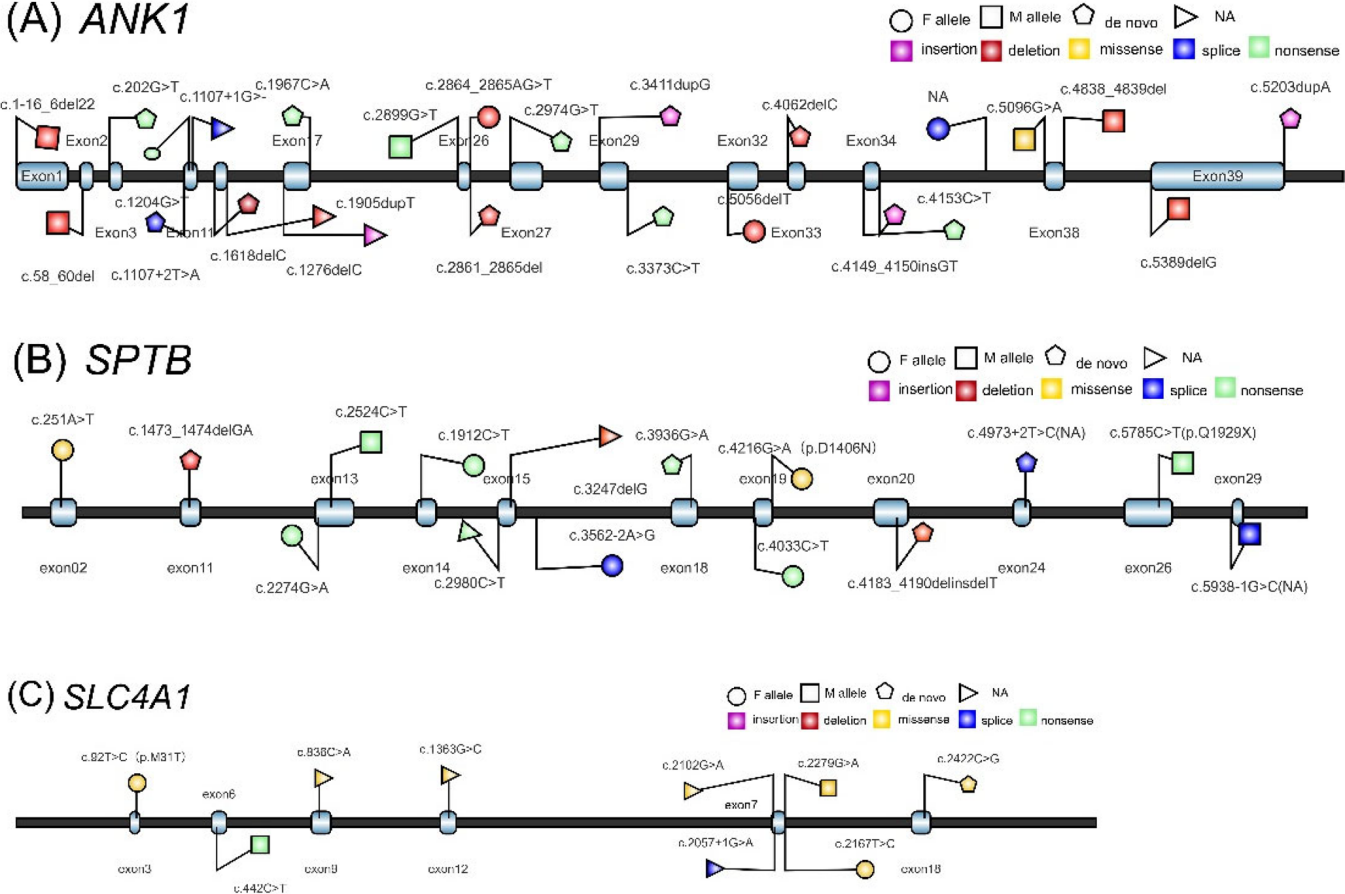
Mutation distribution in *ANK1* (**A**), *SPTB* (**B**), and *SLC4A1* (**C**) genes among 55 HS patients. The schematic was generated using IBS 2.0 software [11]. The figure illustrates the location and type of mutations in each gene. Mutation types are distinguished by symbols and colors : F allele (paternal allele,○), M allele (maternal allele,□), de novo (spontaneous mutation, ), NA (not available, ▽)

Mutations in *SLC4A1* were identified in 9 patients, including 2 with compound heterozygous mutations, who presented with initial hemoglobin levels of 73 g/L and 107 g/L, respectively. *SPTA1* mutations were detected in 2 patients (3.6%), comprising one with compound heterozygous mutations and one with a heterozygous mutation; their corresponding initial Hb levels were 49 g/L and 104 g/L. Additionally, in the present cohort, two patients carried variants in two different HS-associated genes, representing mixed genotypes. One patient harbored variants in *ANK1* (c.2884 C > A, p.P962T; novel, VUS) and *SPTB* (c.3175delG, p.E1059fs; novel, pathogenic), with an initial hemoglobin level of 87 g/L. The other patient carried variants in *ANK1*(c.2174 C > T, p.A725V; novel, VUS) and *SLC4A1* (c.2023 C > T, p.F675L; novel, VUS), with an initial hemoglobin level of 73 g/L. In the first case, the presence of a clearly pathogenic *SPTB* variant likely explains the HS phenotype, suggesting dominant inheritance of the *SPTB* mutation, whereas the contribution of the co-occurring *ANK1* VUS remains uncertain. In the second case, both variants were classified as variants of uncertain significance, and the absence of familial segregation studies precludes determination of whether the phenotype results from digenic inheritance or a single dominant variant. Figure 1 shows the mutation distribution in *ANK1* (A), *SPTB* (B), and *SLC4A1* (C) genes among the 55 HS patients.

### Comparative analysis of main clinical indicators in patients of different ages

To evaluate age-related phenotypic variations in hereditary spherocytosis (HS), the cohort was stratified into two groups based on the age at diagnosis: ≤3 years (*n* = 23) and > 3 years (*n* = 32). Significant intergroup differences were observed in red blood cell (RBC) count, hemoglobin (Hb) levels, absolute reticulocyte counts, mean corpuscular volume (MCV), hematocrit (HCT), and the prevalence of splenomegaly. Specifically, patients in the ≤ 3 years group exhibited significantly more severe anemia, with a mean Hb level of 73.17 ± 16.41 g/L compared to 94.44 ± 16.98 g/L in the > 3 years group (*P <* 0.001), alongside lower RBC counts (2.50 ± 0.69 vs. 3.11 ± 0.45 × 10¹²/L; *P* = 0.001). Notably, while the reticulocyte percentage did not differ significantly between groups, the absolute reticulocyte count was markedly lower in the younger cohort (0.21 ± 0.09 vs. 0.34 ± 0.13 × 10¹²/L; *P <* 0.001). MCV was also significantly reduced in patients ≤ 3 years (84.69 ± 6.29 vs. 90.57 ± 8.13 fL; *P* = 0.005). Regarding clinical complications, splenomegaly was far more prevalent in the ≤ 3 years group (60.9% vs. 12.5%; *P <* 0.001) (Table 4). Conversely, no statistically significant differences were found regarding sex distribution, MCHC, bilirubin levels, or cholelithiasis. These findings suggest that earlier onset of HS is associated with a more severe hematological phenotype and a higher incidence of splenomegaly.

**Table 4:** Comparative analysis of clinical manifestations among HS patients of different age groups

Parameter	Unit	≤ 3 Years (*n* = 23) Mean ± SD or Median [IQR]	> 3 Years (*n* = 32) Mean ± SD or Median [IQR]	Normal Reference Range^a^	*P*-value
Age	years	0.33 [0.16*-*1.00]	16.50 [9.25*–*28.75]	—	—
Sex Ratio (M/F)	—	0.92	0.78	—	0.765
RBC	×10^12^/L	2.50 ± 0.69 (1.28*–*4.26)	3.11 ± 0.45 (2.31*–*3.96)	4.0*-*5.5 (Adults) 3.8*-*5.0 (Children)	0.001
Hemoglobin (Hb)	g/L	73.17 ± 16.41 (49*–*101)	94.44 ± 16.98 (57*–*127)	110*–*160 (Age-dependent)	< 0.001
Reticulocytes (Ret)	%	8.97 ± 3.85 (2.84*–*16.3)	10.56 ± 4.81 (1.90*-*22.13)	0.5*–*1.5	0.197
Absolute Ret Count	×10^12^/L	0.21 ± 0.09 (0.08*–*0.44)	0.34 ± 0.13 (0.12*–*0.64)	0.02*–*0.08	< 0.001
MCV	fL	84.69 ± 6.29 (70.4*–*98.4)	90.57 ± 8.13 (76*-*105.8)	75*–*87 (Children) 80*–*100 (Adults)	0.005
MCHC	g/L	342.96 ± 20.46 (294*–*389)	334.53 ± 16.03 (296*–*363)	320*–*360	0.093
Hematocrit (HCT)	%	21.20 [16.9*–*25.5]	28.00 [26.00*-*31.25]	36*–*46 (Age-dependent)	< 0.001
Total Bilirubin (T-Bil)	µmol/L	56.30 [32.40*–*88.80]	67.50 [57.85*-*102.95]	3.4*–*20.5	0.116
Indirect Bilirubin (I-Bil)	µmol/L	49.80 [25.70*–*82.80]	61.40 [49.08*–*87.35]	< 17.1	0.172
Splenomegaly	n (%)	14 (60.9%)	4 (12.5%)	--	< 0.001
Cholecystolithiasis	n (%)	0 (0%)	1 (3.1%)	Rare in children	—

*HS* hereditary spherocytosis, *RBC* red blood cell, *Hb* hemoglobin, *MCV* mean corpuscular volume, *MCHC* mean corpuscular hemoglobin concentration, *HCT* hematocrit, *T-Bil* total bilirubin, *I-Bil* indirect bilirubin, *SD* standard deviation, *IQR* interquartile range

Data Presentation: Continuous variables are presented as mean ± SD (with range) or median [IQR] based on data distribution. Categorical variables are n (%)

a Reference Values Sources: Normal reference ranges were adopted from Henry’s Clinical Diagnosis and Management by Laboratory Methods (24th ed.) and Nathan and Oski’s Hematology of Infancy and Childhood. Ranges vary slightly by age and laboratory; values listed represent general consensus for pediatric and adult populations

### Comparison of clinical indicators among patients with different gene mutation sites

To evaluate potential phenotypic differences among HS genotypes, patients were categorized into four groups according to the mutated gene. Owing to the limited sample sizes in the *SPTA1* and *SLC4A1* groups, only the *ANK1* and *SPTB* groups were compared. No statistically significant differences were observed in peripheral erythrocyte indices or hemolysis-related parameters between the *ANK1* (*n* = 26) and *SPTB* (*n* = 16) groups.

## Discussion

This study characterized the clinical and genetic features of 55 patients with HS from Sichuan, China, revealing age-related phenotypic heterogeneity and a diverse mutational spectrum. A total of 60 variants were identified, of which 46 (76.7%) were novel, underscoring the considerable genetic heterogeneity of HS. These findings expand the known mutational spectrum of the disease and provide valuable data for future genetic research.

Pathogenic mutations were predominantly identified in *ANK1* (28/55, 50.9%) and *SPTB* (17/55, 30.9%), consistent with prior reports of major causative genes [12–13]. Among the novel variants, 26 of 28 (92.9%) were located in *ANK1* and 12 of 17 (70.6%) in *SPTB*. Pathogenic variants were broadly distributed throughout these genes without apparent hotspot regions, consistent with their autosomal dominant inheritance pattern and underscoring the importance of ankyrin structural integrity and spectrin quality for erythrocyte membrane stability.

In the *SLC4A1* group, one patient carried compound heterozygous mutations (c.2102G > A and c.92T > C). The c.92T > C (p.M31T) variant was classified as likely benign according to ACMG guidelines, whereas the c.2102G > A (p.G701D) variant is a known pathogenic mutation. This case highlights the importance of comprehensive variant interpretation in patients with atypical presentations. These novel mutations not only provide targets for functional studies but also demonstrate that NGS enhances the detection of pathogenic variants—particularly in cases with atypical presentations or no family history—thereby reducing the likelihood of missing rare variants during diagnosis.

Our study highlights a distinct age-dependent phenotypic heterogeneity in HS. Patients diagnosed at ≤ 3 years of age presented with significantly more severe anemia compared to those diagnosed later (> 3 years), evidenced by lower hemoglobin levels (73.17 ± 16.41 g/L vs. 94.44 ± 16.98 g/L; *P <* 0.001) and reduced RBC counts (2.50 ± 0.69 vs. 3.11 ± 0.45 × 10¹²/L; *P* = 0.001). Moreover, splenomegaly was significantly more frequent in the younger cohort (60.9% vs. 3.1%; *P <* 0.001). Stratified analysis by age further indicated that earlier disease onset correlates with inadequate compensatory erythropoiesis, reflected by lower absolute reticulocyte counts in infants and young children (0.21 ± 0.09 vs. 0.34 ± 0.13 × 10¹²/L; *P <* 0.001). This observation aligns with previous reports suggesting that infants with HS often exhibit limited bone marrow compensatory capacity relative to the degree of hemolysis [14]. Potential mechanisms include postnatal maturation of splenic function, which enhances erythrocyte filtration and phagocytosis, thereby exacerbating anemia in early life. Additionally, the high prevalence of splenomegaly in the ≤ 3 years group may be attributed to extramedullary hematopoiesis driven by severe anemia, particularly during concurrent infections. These findings underscore the necessity for close monitoring of hemoglobin levels and splenic size in young HS patients. Older patients who require frequent transfusions or exhibit growth retardation should be rigorously evaluated for splenectomy.

In 2008, Iolascon and Avvisati proposed a correlation between genotype and phenotype in HS, suggesting that biochemical and genetic variations could explain the clinical heterogeneity observed in the disease [15]. In the present cohort, no significant differences were found in hemoglobin levels, reticulocyte counts, or bilirubin levels between patients with *ANK1* and *SPTB* mutations. However, moderate-to-severe anemia was observed in 65.4% (17/26) of patients with *ANK1* mutations and 56.3% (9/16) of those with *SPTB* mutations, suggesting that both genotypes are associated with substantial disease burden. Among the 9 patients with *SLC4A1* mutations, 55.6% (5/9) presented with moderate-to-severe anemia.

**Fig. 2:**
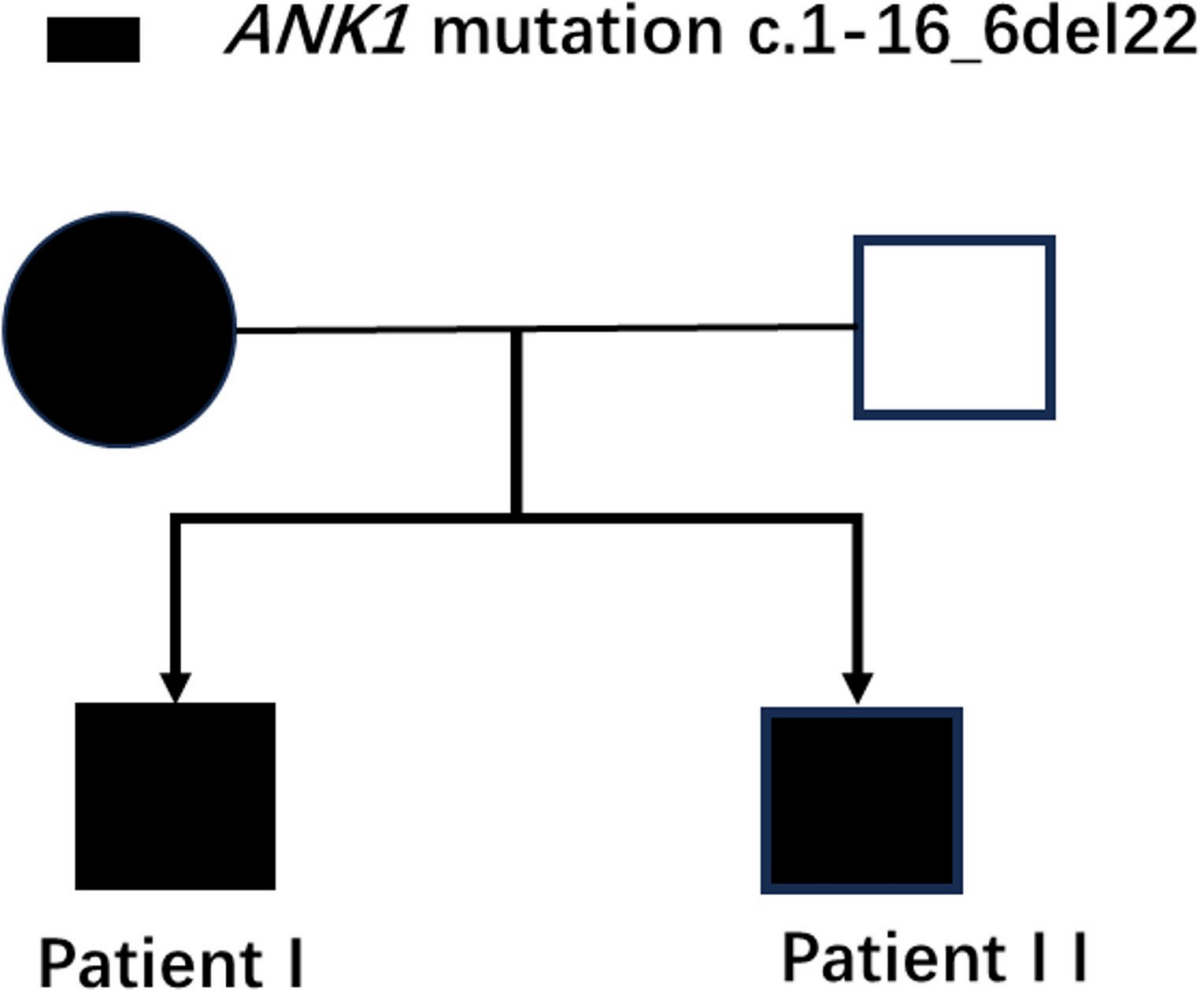
Genotype-phenotype correlation of ANK1 mutation (c.1–16_6del22) in a HS-affected family. Pedigree of a family with HS harboring the pathogenic *ANK1* deletion mutation c.1–16_6del22. Two siblings (Patient I and Patient II) inherited the mutation, while their mother (carrier) also carried the mutation. Clinical phenotypes and management outcomes are detailed below: Patient I (elder sibling): Severe HS phenotype, with hemoglobin (Hb) 49 g/L, reticulocytosis (9%), and severe splenomegaly; underwent splenectomy at 6 years of age. Patient II (younger sibling): Moderate HS phenotype, with moderate anemia (Hb 74 g/L), reticulocytosis (7.3%), and mild splenomegaly; underwent splenectomy at 7 years of age. Mother (mutation carrier): Only mild anemia, no splenomegaly or need for splenectomy.)

In the *ANK1* group, two siblings carrying the same mutation (c.1–16_6del22)—both inherited from their mother (pedigree shown in Fig. 2)—underwent splenectomy. At initial diagnosis, the elder sibling presented with severe anemia (Hb 49 g/L, reticulocytes 9%) and severe splenomegaly, while the younger sibling presented with moderate anemia (Hb 74 g/L, reticulocytes 7.3%) and mild splenomegaly. Their mother, also a mutation carrier, exhibited only mild anemia without splenomegaly and did not require splenectomy. This observation highlights significant intrafamilial phenotypic heterogeneity in HS, wherein identical mutations can lead to divergent clinical severity. Such variability may arise from genetic modifiers, differential gene expression, environmental influences, sex differences, age at presentation, or variations in genetic background [16–19]. Notably, the mother’s milder phenotype in adulthood is consistent with the known natural history of HS, in which anemia often improves with age due to enhanced compensatory erythropoiesis. Additionally, potential coexisting genetic factors from the paternal lineage that could influence hemolysis were excluded through familial segregation analysis. Further studies are warranted to elucidate the underlying mechanisms of phenotypic variability, which may improve the diagnosis and management of HS.

Among the 46 novel mutations identified, 21 were classified as variants of uncertain significance (VUS) according to ACMG guidelines, indicating that their clinical relevance remains unclear and requires further validation through longitudinal follow-up, familial segregation studies, bioinformatic analyses, and functional assays. The limited sample size, particularly in the *SPTA1* group (*n* = 2), may have led to an underestimation of the prevalence of recessive mutations. Furthermore, the absence of quantitative erythrocyte membrane protein electrophoresis data restricted the analysis of correlations between genotype and membrane protein expression. This limitation highlights the need for more comprehensive genetic analyses in future studies of HS.

## Conclusion

In conclusion, this study confirms that *ANK1* and *SPTB* are the predominant pathogenic genes in patients with HS from Sichuan, China. Earlier disease onset was significantly associated with more severe anemia, and 46 novel mutations were identified, substantially expanding the mutational spectrum of HS. Future studies should focus on elucidating the pathogenesis of HS to identify novel targets for precision therapy.

## Supplementary Information

Below is the link to the electronic supplementary material.

Supplementary Material 1

## Data Availability

Data is not publicly available.
