# Supplementary material for: Expanding the genetic spectrum of hereditary spherocytosis: novel mutations and phenotypic heterogeneity from a 55-patient cohort
Source: Ann Hematol. 2026 May 13;105(7):321. doi: 10.1007/s00277-026-07066-1 (PMC13337869; doi:10.1007/s00277-026-07066-1)
Supplement: Supplementary file 1 — Supplementary Material 1 [file 277_2026_7066_MOESM1_ESM.docx]

**Supplementary Table 2. Detailed Characterization of All 60 Identified Variants in Hereditary Spherocytosis Patients.**

**A. *ANK1* Gene Variants (n=28)**

| Site (GRCh37) | Exon | cDNA Change | Protein Change | Type | Zygosity | Inheritance | Status | ACMG Class |
| --- | --- | --- | --- | --- | --- | --- | --- | --- |
| chr8:41655051-41655072 | 1 | c.1-16_6del22^&^ | p.M1_P2delMP | Deletion | Het | Maternal | **Novel** | VUS |
| chr8:41615623 | 2 | c.58_60del | p.20_20del | Deletion | Het | Maternal | **Novel** | VUS |
| chr8:41591515 | 3 | c.202G>T | p.E68* | Nonsense | Het | *De novo* | **Novel** | Pathogenic |
| chr8:41577178 | 11 | c.1107+1G>- | p.? | Splice | Het | NA | **Novel** | Pathogenic |
| chr8:41577177 | 10 | c.1107+2T>A | p.? | Splice | Het | *De novo* | **Novel** | Pathogenic |
| chr8:41575626 | 11 | c.1204G>T | p.E402* | Nonsense | Het | Paternal | **Novel** | Pathogenic |
| chr8:41575151 | 12 | c.1276delC | p.R426Efs*32 | Deletion | Het | NA | **Novel** | Pathogenic |
| chr8:41573253 | NA | c.1618delC | p.L540Cfs*26 | Deletion | Het | *De novo* | **Novel** | Pathogenic |
| chr8:41566388 | 17 | c.1905dupT | p.V636Cfs*17 | Insertion | Het | NA | **Novel** | Pathogenic |
| chr8:41566327 | 17 | c.1967C>A | p.S656* | Nonsense | Het | *De novo* | **Novel** | Pathogenic |
| chr8:41553976 | 26 | c.2861_2865del | p.V954Sfs*11 | Deletion | Het | *De novo* | **Novel** | Pathogenic |
| chr8:41553976 | 26 | c.2864_2865AG>T | p.K955Rfs*10 | Deletion | Het | Paternal | **Novel** | Pathogenic |
| chr8:41553957 | 26 | c.2884C>A | p.P962T | Missense | Het | Paternal | **Novel** | VUS |
| chr8:41553942 | 26 | c.2899G>T | p.E967* | Nonsense | Het | Maternal | Reported | Pathogenic |
| chr8:41552836 | 27 | c.2974G>T | p.E992* | Nonsense | Het | *De novo* | **Novel** | VUS |
| chr8:41551575 | 29 | c.3373C>T | p.Q1125* | Nonsense | Het | *De novo* | **Novel** | Pathogenic |
| chr8:41551536 | 29 | c.3411dupG | p.R1138Kfs*15 | Insertion | Het | *De novo* | **Novel** | Pathogenic |
| chr8:41547787 | 33 | c.4062delC | p.H1354Qfs*6 | Deletion | Het | *De novo* | **Novel** | Pathogenic |
| chr8:41547822 | 34 | c.4149_4150insGT | p.L1384Vfs*6 | Insertion | Het | *De novo* | **Novel** | Pathogenic |
| chr8:41546059 | 34 | c.4153C>T | p.R1385* | Nonsense | Het | *De novo* | Reported | Pathogenic |
| chr8:41543662 | 36 | NA | p.? | Splice | Het | Paternal | **Novel** | Pathogenic |
| chr8:41530129 | 38 | c.4838_4839del | p.P1613Rfs*26 | Deletion | Het | Maternal | **Novel** | Pathogenic |
| chr8:41529912 | 32 | c.5056delT | p.S1686Pfs*4 | Deletion | Het | Paternal | **Novel** | Pathogenic |
| chr8:41529872 | 38 | c.5096G>A | p.R1699K | Missense | Het | Maternal | **Novel** | VUS |
| chr8:42525975 | 39 | c.5203dupA | p.T1735Nfs*15 | Insertion | Het | *De novo* | **Novel** | Pathogenic |
| chr8:41525790 | 39 | c.5389delG | p.V1797Cfs*28 | Deletion | Het | Maternal | **Novel** | VUS |
| chr8:41561914 | 16 | c.2174C>T | p.A725V | Missense | Het | Maternal | **Novel** | VUS |
|  |  |  |  |  |  |  |  |  |

^&:^ ^two were siblings both carrying the^ *^ANK1^*^( c.1-16_6del22 )^

**B. *SPTB* Gene Variants (n=16)**

| Site (GRCh37) | Exon | cDNA Change | Protein Change | Type | Zygosity | Inheritance | Status | ACMG Class |
| --- | --- | --- | --- | --- | --- | --- | --- | --- |
| chr14:65271706 | 2 | c.251A>T | p.D84V | Missense | Het | Paternal | **Novel** | VUS |
| chr14:65262225-65262226 | 11 | c.1473_1474delGA | p.E491Dfs*9 | Deletion | Het | *De novo* | **Novel** | Pathogenic |
| chr14:65260469 | 14 | c.1912C>T | p.R638* | Nonsense | Het | Paternal | Reported | Pathogenic |
| chr14:65260107 | 13 | c.2274G>A | p.W758* | Nonsense | Het | Paternal | **Novel** | Pathogenic |
| chr14:65259857 | 13 | c.2524C>T | p.Q842* | Nonsense | Het | Maternal | **Novel** | Pathogenic |
| chr14:65253703 | 15 | c.2980C>T | p.Q994* | Nonsense | Het | NA | **Novel** | Pathogenic |
| chr14:65253508 | 15 | c.3175delG | p.E1059Kfs*7 | Deletion | Het | Maternal | **Novel** | Pathogenic |
| chr14:65253436 | 15 | c.3247delG | p.A1083Lfs*16 | Deletion | Het | NA | **Novel** | Pathogenic |
| chr14:65252671 | NA | c.3562-2A>G | p.? | Splice | Het | Paternal | Reported | Pathogenic |
| chr14:65251031 | 18 | c.3936G>A | p.W1312* | Nonsense | Het | *De novo* | **Novel** | VUS |
| chr14:65249241 | 19 | c.4033C>T | p.Q1345* | Nonsense | Het | Paternal | **Novel** | Pathogenic |
| chr14:65249058 | 19 | c.4216G>A | p.D1406N | Missense | Het | Paternal | **Novel** | VUS |
| chr14:65249091 | 20 | c.4183_4190delinsdelT | p.I1395Lfs*32 | Indel | Het | *De novo* | **Novel** | Pathogenic |
| chr14:65241113 | 24 (intron) | c.4973+2T>C | p.? | Splice | Het | *De novo* | **Novel** | Pathogenic |
| chr14:65237616 | 26 | c.5785C>T | p.Q1929* | Nonsense | Het | Maternal | **Novel** | VUS |
| chr14:65235837 | 29 | c.5938-1G>C | p.? | Splice | Het | Maternal | **Novel** | VUS |

**C. *SLC4A1* Gene Variants (n=12)**

| Site (GRCh37) | Exon | cDNA Change | Protein Change | Type | Zygosity | Inheritance | Status | ACMG Class |
| --- | --- | --- | --- | --- | --- | --- | --- | --- |
| chr17:42336571 | 9 | c.836C>A | p.T279N | Missense | Het | NA | **Novel** | VUS |
| chr17:42335095 | 12 | c.1363G>C | p.G455R | Missense | Het | NA | **Novel** | VUS |
| chr17:42330695 | 17 | c.2102G>A | p.G701D | Missense | Het | NA | Reported | Pathogenic |
| chr17:42331863 | 17 | c.2057+1G>A | p.? | Splice | Het | NA | Reported | VUS |
| chr17:42330630 | 17 | c.2167T>C | p.W723R | Missense | Het | Paternal | **Novel** | VUS |
| chr17:42330518 | 17 | c.2279G>A | p.R760Q | Missense | Het | Maternal | **Novel** | Pathogenic |
| chr17:42328846 | 18 | c.2422C>G | p.R808G | Missense | Het | *De novo* | **Novel** | Pathogenic |
| chr17:42340018 | 3 | c.92T>C | p.M31T | Missense | Het | Paternal | Reported | Likely Benign |
| chr17:42337815 | 6 | c.442C>T | p.Q148* | Nonsense | Het | Maternal | **Novel** | Pathogenic |
| chr17:42331898 | 19 | c.2023C>T | p.F675L | Missense | Het | Maternal | **Novel** | VUS |
| chr17:42337815 chr17:42330695 | 6/17 | c.442C>T c.2102G>A | p.Q148* p.G701D | Nonsense Missense | D-het | Mat/Pat | **Novel** / Reported | Pathogenic / Pathogenic |
| chr17:42330695 chr17:42340018 | 17/3 | c.2102G>A c.92T>C | p.G701D p.M31T | Missense Missense | D-het | Pat/Pat | Reported / Reported | Pathogenic / Likel |

**D. Other Genes and Compound Heterozygotes (4 Cases)**

| Gene(s) | Site (GRCh37) | Exon | cDNA Change | Protein Change | Type | Zygosity | Inheritance | Status | ACMG Class |
| --- | --- | --- | --- | --- | --- | --- | --- | --- | --- |
| *SPTA1* | chr1:158627401 | 19 | c.2671C>T | p.R891* | Nonsense | Het | Paternal | Reported | Pathogenic |
| *SPTA1* / *SPTA1* | chr1:158622298 chr1:158623071 | 23/22 | c.3334G>T c.3181G>C | p.D1112Y p.E1061Q | Missense Missense | D-het | Pat/Mat | **Novel** / **Novel** | VUS / VUS |
| *ANK1* / *SPTB* | chr8:41553957 chr14:65253508 | 26/15 | c.2884C>A c.3175delG | p.P962T p.E1059Kfs*7 | Missense Deletion | D-het | Pat/Mat | **Novel** / **Novel** | VUS / Pathogenic |
| *ANK1* / *SLC4A1* | chr8:41561914 chr17:42331898 | 16/19 | c.2174C>T c.2023C>T | p.A725V p.F675L | Missense Missense | D-het | Mat/Mat | **Novel** / **Novel** | VUS / VUS |
